# Predominance of Single Prophage Carrying a CRISPR/*cas* System in “*Candidatus* Liberibacter asiaticus” Strains in Southern China

**DOI:** 10.1371/journal.pone.0146422

**Published:** 2016-01-07

**Authors:** Zheng Zheng, Minli Bao, Fengnian Wu, Jianchi Chen, Xiaoling Deng

**Affiliations:** 1 Guangdong Province Key Laboratory of Microbial Signals and Disease Control, College of Agriculture, South China Agricultural University, Guangzhou, Peoples' Republic of China; 2 San Joaquin Valley Agricultural Sciences Center, United States Department of Agriculture–Agricultural Research Service, Parlier, California, United States of America; Key Laboratory of Horticultural Plant Biology (MOE), CHINA

## Abstract

“*Candidatus* Liberibacter asiaticus” (CLas) is an uncultureable α-proteobacterium associated with citrus Huanglongbing (HLB, yellow shoot disease), a highly destructive disease affecting citrus production worldwide. HLB was observed in Guangdong Province of China over a hundred years ago and remains endemic there. Little is known about CLas biology due to its uncultureable nature. This study began with the genome sequence analysis of CLas Strain A4 from Guangdong in the prophage region. Within the two currently known prophage types, Type 1 (SC1-like) and Type 2 (SC2-like), A4 genome contained only a Type 2 prophage, CGdP2, namely. An analysis on CLas strains collected in Guangdong showed that Type 2 prophage dominated the bacterial population (82.6%, 71/86). An extended survey covering five provinces in southern China also revealed the predominance of single prophage (Type 1 or Type 2) in the CLas population (90.4%, 169/187). CLas strains with two and no prophage types accounted for 7.2% and 2.8%, respectively. *In silico* analyses on CGdP2 identified a CRISPR (clustered regularly interspaced short palindromic repeats)/*cas* (CRISPR-associated protein genes) system, consisting of four 22 bp repeats, three 23 bp spacers and 9 predicted *cas*. Similar CRISPR/*cas* systems were detected in all 10 published CLas prophages as well as 13 CLas field strains in southern China. Both Type 1 and Type 2 prophages shared almost identical sequences in spacer 1 and 3 but not spacer 2. Considering that the function of a CRISPR/*cas* system was to destroy invading DNA, it was hypothesized that a pre-established CLas prophage could use its CRISPR/*cas* system guided by spacer 1 and/or 3 to defeat the invasion of the other phage/prophage. This hypothesis explained the predominance of single prophage type in the CLas population in southern China. This is the first report of CRISPR/*cas* system in the “*Ca*. Liberibacter” genera.

## Introduction

“*Candidatus* Liberibacter asiaticus” (CLas) is associated with citrus Huanglongbing (HLB), a highly destructive disease in citrus production worldwide [1]. In China, HLB was reported in Pearl River Delta area of Guangdong Province in 1919 [2]. Observations by growers can be dated back to the late 1880s in Chaoshan area of Guangdong, where the name Huanglongbing (yellow shoot disease) was derived [3]. The infectious nature of HLB was recognized in early research [4,5]. However, efforts to search for HLB causal agent were not successful until more recently [6]. In 1994, HLB was associated with CLas, represented by Strain “Poona” from India [7]. Two years later, CLas was confirmed to associate with HLB in Guangdong [8,9].

The pathogen status of CLas in HLB is putatively established on repeated findings of an association between symptoms and bacterium presence. However, Koch’s postulates have not been completed because CLas is non-culturable *in vitro*. For over a decade, CLas research in China was limited to bacterial detection and population evaluation based on conserved genomic loci [8–12]. Aided by the next generation sequencing (NGS) technology, the genome of a Florida CLas (Strain Psy62) was sequenced [13]. A hypervariable locus (CLIBASIA_01645) in the bacterial chromosome was identified and successfully differentiated the CLas populations between Guangdong and Florida [14]. This locus was further used to characterize CLas populations from Brazil [15], the Caribbean [16], China [17], and India [18].

Another significant discovery from CLas genome sequence analyses is the identification of prophage, the lysogenic form of a phage with its DNA inserted into the bacterial chromosome. The Psy62 genome was found to harbor a prophage [13], later named as FP1, along with another prophage FP2 [19]. Two prophages, SC1 and SC2, their circular replication forms, and possible phage particles were reported in the Florida strain, UF506 [20]. Several whole genome sequences (both complete and draft versions) of CLas are now available [21–25]. All but a Japanese strain [25] were found to harbor prophages. There are currently two known types of CLas prophages, Type 1 (SC1-like) and Type 2 (SC2-like). Type 1 and Type 2 prophages are structurally similar and reported to be connected in tandem in CLas chromosome [20,21]. A recent analysis, however, revealed a CLas strain with single prophage [24]. Little information is available about the biological roles and interactions between the two prophages. Prophages/phages are of high interest because of their lytic property that could be used for CLas control, and their influence on CLas behaviors, such as culturability [25] and eliciting host defense [26,27].

Along with available whole genome sequences, the CRISPR (clustered regularly interspaced short palindromic repeats)/*cas* (CRISPR associated protein genes) systems were found in the genomes of almost all archaea and about half of bacterial species [28,29]. Bacteria acquire resistance to foreign DNA by incorporation of short transcribed nucleotide sequences into regions of CRISPR called spacers. Following transcription and processing of these loci, the CRISPR RNAs (crRNAs) guide the Cas proteins to complementary invading nucleic acid, resulting in targeted destruction. CRISPR are usually located adjacent to the *cas* genes [28]. CRISPR/*cas* systems are believed to be frequently exchanged via horizontal gene transfer [30]. There have not been reports on the presence of CRISPR/*cas* system in any member of CLas.

A draft genome sequence of CLas strain A4 from plant (periwinkle) host in Guangdong of China was published [22], which is used to represent CLas strains from the historical HLB region. In this study, we re-assembled and evaluated the A4 sequence with a focus on the prophage region. Sequence analyses found that strain A4 harbored only a single prophage carrying a CRISPR/*cas* system. An extensive survey revealed the predominance of single prophage in the CLas population in southern China, which could be explained by the predicted function of the CRSPR/*cas* system.

## Materials and Methods

### A4 and other CLas strains

**Fig 1:**
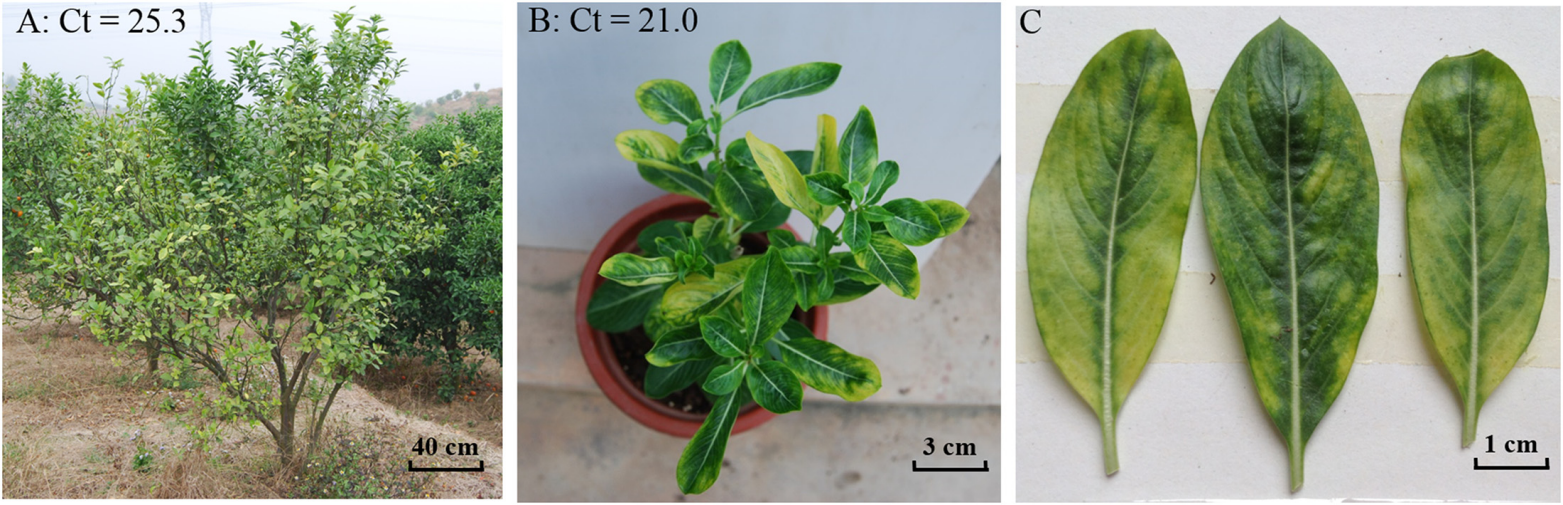
“*Candidatus* Liberibacter asiaticus” strain A4 in two plant hosts in Guangdong, China. (A) A Huanglongbing (HLB) symptomatic tree of *Citrus reticulata* cultivar “Shatangju” in Sihui City, Guangdong, China. (B) Symptomatic periwinkle plant infected by “*Ca*. L. asiaticus” via dodder transmission from citrus. The CLas strain was designated as A4 and maintained and propagated in a screenhouse through grafting. (C) Symptomatic periwinkle leaves used to extract DNA for genomic study. Increase of “*Ca*. L. asiaticus” titer from citrus to periwinkle is indicated by the decrease of Ct values using the PCR procedure described by Li et al. [31].

**Fig 2:**
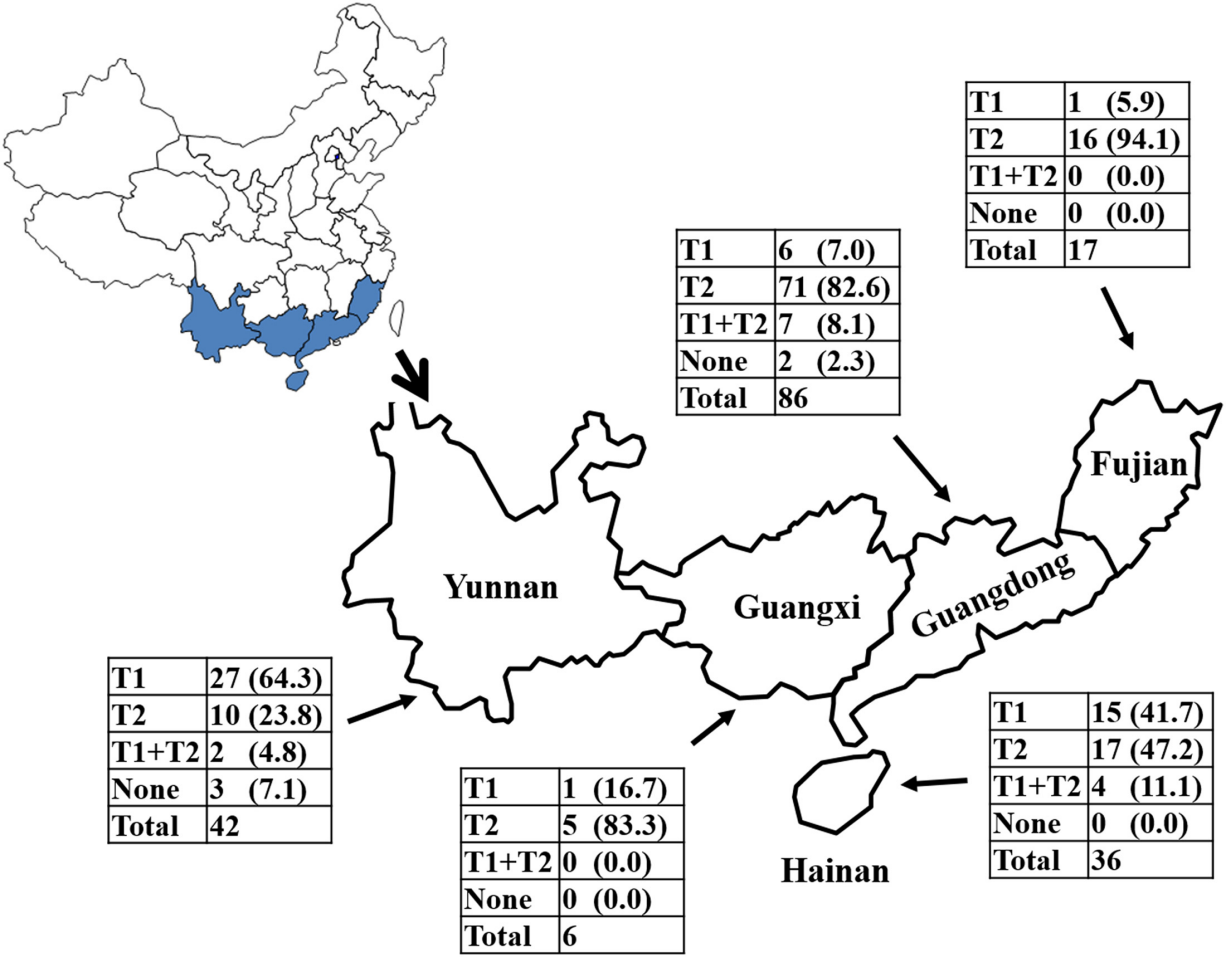
Distribution of prophage types of “*Candidatus* Liberibacteria asiaticus” in five provinces in southern China. A map of China is shown on the upper left. The five provinces where “*Ca*. L. asiaticus” strains were collected are outlined. Distribution of prophage types in each province is summarized in a table. T1 = Type 1; T2 = Type 2. The numbers in parentheses are calculated percentage.

CLas strain A4 originated from a collection in an HLB outbreak in Sihui City of Guangdong Province, People’s Republic of China in December of 2005 (Fig 1A). The bacterium was first grafted on a healthy mandarin citrus (*Citrus reticulata* Blanco), cultivar “Shatangju”, and transmitted to periwinkle (*Catharanthus roseus* (L.)G. Don.) via dodder (*Cuscuta campestris* Yunck). CLas was monitored by PCR with primer set OI1-OI2c [7] and quantified by the procedure of Li et al. [31] with primer set HLBasf/HLBasr (Fig 1A and 1B). Strain A4 was maintained, propagated through grafting, and used as DNA source for sequence evaluation. Other CLas strains used in this study were collected from HLB affected citrus trees in five provinces in southern China (Fig 2). DNA was extracted following the procedure described previously [17]. Infection of CLas was confirmed by the procedure described by Li et al.[31]. A DNA sample from a single tree, or a single Asian citrus psyllid (*Diaphorina citri* Kuwayama), the vector of CLas, was considered as a CLas strain. For citrus origin, total plant DNA was extracted by E. Z. N. A.HP Plant DNA Kit (OMEGA Bio-Tek Co., Guangdong, China) using 200 mg of leaf midribs from three citrus leaves collected from the same branch of HLB-infected tree. For the Asian citrus Psyllid (*Diaphorina citri* Kuwayama), DNA was extracted with TIANamp Genomic DNA Kit (Tiangen Biotech Co., Beijiang, China) from single insects following the manufacturer’s protocol.

### Re-evaluation of A4 genome sequence

A brief description of strain A4 genome sequencing using Illumina MiSeq platform with Strain Psy62 genome sequence (CP001677.5) [13] as a reference was published previously [22]. Because the Psy62 genome sequence did not include prophage FP2 (a SC2 homolog), the A4 genome sequence was reassembled by including SC2 sequence (NC_019550.1) as a reference following the same procedure [22], mainly involving identification of CLas reads based on reference sequences with standalone BLAST [32], read collection with Perl scripts, and a combination of *de novo* assembly with Velvet 1.2.10 [33] and referenced assembling with CLC Genomic Workbench 7.5.

For gap closure, primers were designed using Primers 3 software [34] based on contig sequences from assembly results. PCR was performed following standard procedures. Amplicons generated from these primers were cloned in *pEASY-*T1 plasmid (TransGen Biotech, Beijing, China) or directly sequenced by Sanger’s method. Sequences were assembled with SeqMan software under the DNASTAR Lasergene suit (http://www.dnastar.com). Genome annotation was conducted using the RAST server (http://rast.nmpdr.org) [35].

### Genome sequence comparisons

Whole genome sequences of CLas strains and related prophages were downloaded from National Center for Biotechnology Information (NCBI, http://www.ncbi.nlm.nih.gov/) (Table 1). Standalone BLAST software was used for pair-wise genome comparison. Multiple sequence alignment was performed on the Clustal Omega Server (http://www.ebi.ac.uk/Tools/msa/clustalo) [36].

**Table 1:** General information of 8 published genome sequences of “*Candidatus* Liberibacter asiaticus” strains and their prophages.

Strain	Accession	Origin	Number of prophage	Size of Type 1 prophage (bp) /Name/Accession	Size of Type 2 prophage (bp) /Name/Accession	Reference
**UF506**	**HQ377374.1**	**Florida**	**2**	**40,048 / SC1 / NC_019549.1**	**38,997 / SC2 / NC_019550.1**	[20]
**Psy62**	**CP001677.5**	**Florida**	**2**	**39,467 / FP1/ na**^a^	**38,552 / FP2 / JF773396.1**	[13]^b^
**Gxpsy**	**CP004005.1**	**Guangxi, China**	**2**	**37,794 / nn**^c^ **/ na**	**40,277 / nn**^c^**/ na**	[21]^d^
**Ishi-1**	**AP014595.1**	**Japan**	**0**	-	-	[25]
**A4**	**CP010804**	**Guangdong, China**	**1**	-	**38,918 / CGdP2 / na**	[22]
**HHCA**	**JMIL02000000**	**Hacienda Heights, CA**	**1**	-	**38,945 / nn / na**	[23]^e^
**FL17**	**JWHA01000000**	**Central Florida**	**1**	**39,143 / nn / na**	-	[24]^f^
**YCpsy**	**LIIM01000000**	**Guangdong, China**	**1**	**39,304 / nn / na**	-	[44]^g^

^a^ na, No accession number available.

^b^ The FP1 sequence was identified from Psy62 genome sequence based on similarity to SC1.

^c^ nn, no name assigned to the prophage.

^defg^ The prophage sequences were identified based on similarity to SC1 or SC2.

### Evaluating and defining prophage types

Zhang et al. [20] reported two CLas prophages, SC1 and SC2, and research so far has shown that all known CLas prophages were related to either SC1 or SC2. Therefore, two prophage types, Type 1 and Type 2, were designated anchoring similarity to SC1 or SC2, respectively. For strains with MiSeq data such as A4, or published sequence data (Table 1), the mapping method was used. Prophage type was determined by mapping the MiSeq sequence reads, or the prophage sequences, to SC1 and SC2 using CLC genomic workbench version 7.5. For field collected samples, the PCR method was used. Specific PCR primers were designed by comparing the sequences between SC1 and SC2. Eight loci/regions unique to SC1 and SC2 after alignment between the two sequences were selected. Primer sets were designed using Primer 3 software [34]. Primer sequences and related information are listed in Table 2. Prophage type was determined by the success of PCR experiments yielding expected amplicons from at least 6 out of the 8 specific primer sets. CLas strains from five provinces (Yunnan, Guangxi, Hainan, Guangdong and Fujian) in southern China were used for distribution analysis of different prophage type (Fig 2). The percentage of CLas strains with different type of prophage from each province were calculated based on the PCR result.

**Table 2:** General information of primers specific to Type 1 or Type 2 prophage of “*Candidatus* Liberibacter asiaticus” based on comparisons of prophage sequences between SC1 and SC2.

Code	Primer set (F/R)	Sequence (5’ → 3’) set (F/R)	Amplicon size (bp)	Location	Locus name	Putative function	Prophage Type
**1**	**SC1-1F/SC1-1R**	**ATCCTTTGACAGTGAGGCCA/CTCGTGAGGTTCTTGAGGGT**	**1,025**	**4854–5879**	**SC1_gp030**	**Structural protein**	**1**
**2**	**SC1-2F/SC1-2R**	**TGGCTCGGGTTCAGGTAAAT/AAGGGCGACGCATGTATTTC**	**975**	**6236–7211**	**SC1_gp035**	**Endolysin**	**1**
**3**	**SC1-3F/SC1-3R**	**CTCACTGCGTCTTGATTCGG/CGAACGAGCGGTATGTTTGT**	**866**	**9296–10162**	**SC1_gp050**	**Phage-related protein**	**1**
**4**	**SC1-4F/SC1-4R**	**GCACCTAAAATAGCCGGCTC/GGGGTTGAGGCGGTATATCA**	**954**	**10589–11543**	**SC1_gp060**	**Hypothetical protein**	**1**
**5**	**SC1-5F/SC1-5R**	**TCGTAGGATCGTAACACCCG/CGGTGGTTATGCGTTACTGG**	**888**	**14502–15390**	**SC1_gp080**	**Phage-related protein**	**1**
**6**	**SC1-6F/SC1-6R**	**GTGGTGTTGAAGGTAGGGGA/TCGATGGAAAAGACCCGTGA**	**892**	**17859–18751**	**SC1_gp095**	**Glutathione peroxidase**	**1**
**7**	**SC1-7F/SC1-7R**	**CGATCTGGCGTCCTCCTTAT/GCGAGCCTTATCAACCACAG**	**918**	**19629–20547**	**SC1_gp110**	**Holin**	**1**
**8**	**SC1-8F/SC1-8R**	**GGGAGGGTTTTACGAATGGC/TGCCTCGCTCAAAGACCTTA**	**868**	**3379–4247**	**SC1_gp030**	**Structural protein**	**1**
**9**	**SC2-1F/SC2-1R**	**GCACCTCTCGCATACCAAAG/GTCGGTGGTTTTACTCGCAA**	**807**	**1891–2717**	**SC2_gp030**	**Structural protein**	**2**
**10**	**SC2-2F/SC2-2R**	**ACCCTCGCACCATCATGTTA/TCGTCTTGATTGGGCAGAGT**	**813**	**2741–3554**	**SC2_gp030**	**Structural protein**	**2**
**11**	**SC2-3F/SC2-3R**	**ACAGTTAAGAGCCACGGTGA/AAGACGTGGGTGTTATGGGT**	**918**	**4220–5138**	**SC2_gp040**	**Phage-related protein**	**2**
**12**	**SC2-4F/SC2-4R**	**AACATCCACCTGTCCCTCTG/ACGTCTCGGTGGCTTAAAGA**	**978**	**5237–6215**	**SC2_gp045**	**Phage-related protein**	**2**
**13**	**SC2-5F/SC2-5R**	**CCCATGCGTCCTGTCTAGAA/TAGTATTGCCGTTTCCCCGA**	**951**	**9429–10380**	**SC2_gp075**	**Exodeoxyribonuclease**	**2**
**14**	**SC2-6F/SC2-6R**	**CTTTTCCCTTCACGTCGAGC/AAAGGCGTTAAACCCAGCAG**	**885**	**14077–14962**	**SC2_gp100**	**Glutathione peroxidase**	**2**
**15**	**SC2-7F/SC2-7R**	**CTGCTGGGTTTAACGCCTTT/ATGAGGCTTTGGACACTGGT**	**942**	**14962–15904**	**SC2_gp105**	**Head-to-tail joining protein**	**2**
**16**	**SC2-8F/SC2-8R**	**CATAGCCCCTCCCTCAGTTC/GCGGGAGTCAAGATAACACC**	**795**	**34800–35595**	**SC2_gp240**	**Trimeric autotransporter adhesin**	**2**

^a^ na, No accession number available.

^b^ The FP1 sequence was identified from Psy62 genome sequence based on similarity to SC1.

^c^ nn, no name assigned to the prophage.

^defg^ The prophage sequences were identified based on similarity to SC1 or SC2.

### CRISPR/*cas* analyses

A CRISPR/*cas* system was defined by the simultaneous presence of a CRISPR array and *cas* genes in the nearby vicinity [28]. Candidate CRISPR repeats array were detected by CRISPR Recognition Tool [37]. Alignment of CRISPR repeat sequences was performed on the Clustal Omega Server [36] and viewed by Jalview [38]. The secondary structure of CRISPR repeat transcript (represented by DNA sequences) was predicted by Quikfold on DINAMelt web server with default setting [39]. To check for possible sequence origins, spacers were used as queries for BLASTn against nucleotide sequence database including the virus database in GenBank (version 1.1).

Genes or ORFs adjacent to CRISPR repeat array were selected and used as queries to search for the presence of *cas* gene in Conserved Domain Database (CDD, version 3.13) that included the most updated collection of published *cas* genes [40]. Once a candidate CRISPR/*cas* system was identified, the sequence in the vicinity was downloaded and used as query to search for homologs in other published CLas genomes (Table 1) using BLASTn. Variations of the CRISPR locus among known CLas genomes were analyzed through multiple sequence alignment by Clustal Omega [36]. Phylogenetic analyses were performed on MEGA 6.0 [41].

To investigate variations of the CRISPR array, additional CLas strains were collected from southern China. Prophage types were determined by the PCR method (Table 2). The CRISPR regions were PCR amplified with primer set CRIF/CRIR (CTCAGCTTTTGTCATGCCCA / AGGAAGACAATATCGCCCGT). Amplicons were sequenced by Sanger’s method.

## Results and Discussion

### Re-evaluation of A4 genome sequence

To bypass the *in vitro* culture barrier, the *in planta* culture system was used to supply Strain A4 DNA continuously. As shown in Fig 1, periwinkle was an effective host for CLas enrichment. A drop of over 4 Ct value (25.3 in citrus vs. 21.0 in periwinkle) was achieved. Further CLas DNA enrichment procedures were described previously [22]. Based on the number of MiSeq reads, the CLas/periwinkle DNA ratio was about 0.02 or 1:50 (636,810 CLas-reads vs. 32,130,744 non-CLas reads), rather than the possible 1:1,000 [31]. Over 20,000 bp were re-sequenced from PCR amplicons with a total of 225 primer sets to improve quality of the previous version of A4 genome sequence. The new version of A4 genome (CP010804) consisted of 1,233,514 bp, with the average GC content of 36.4%, 1,187 ORFs, and 53 RNA genes.

### Special features of A4 genome

Comparison of whole genome sequences between strain A4 and selected strains (Psy62, Ishi-1 and gxpsy) from different geographical origins showed limited variations in the chromosomal region, mostly single nucleotide polymorphisms (SNPs) and indels (insertions/deletions) including tandem repeat variations reported previously [14,17]. A feature of particular interest was the presence of a single prophage. Among the 636,810 CLas reads (mean length = 250 bp) from the MiSeq data, no reads were matched to Psy62 genome at several regions corresponding to prophage FP1 (homolog of SC1). A visualization of A4 MiSeq reads mapped to SC1 and SC2 were performed by CLC genomic workbench (S1 Fig). A4 reads covered 57% of SC1 and 100% of SC2, indicating the presence of a Type 2 prophage, designated as CGdP2, in the A4 genome.

**Fig 3:**
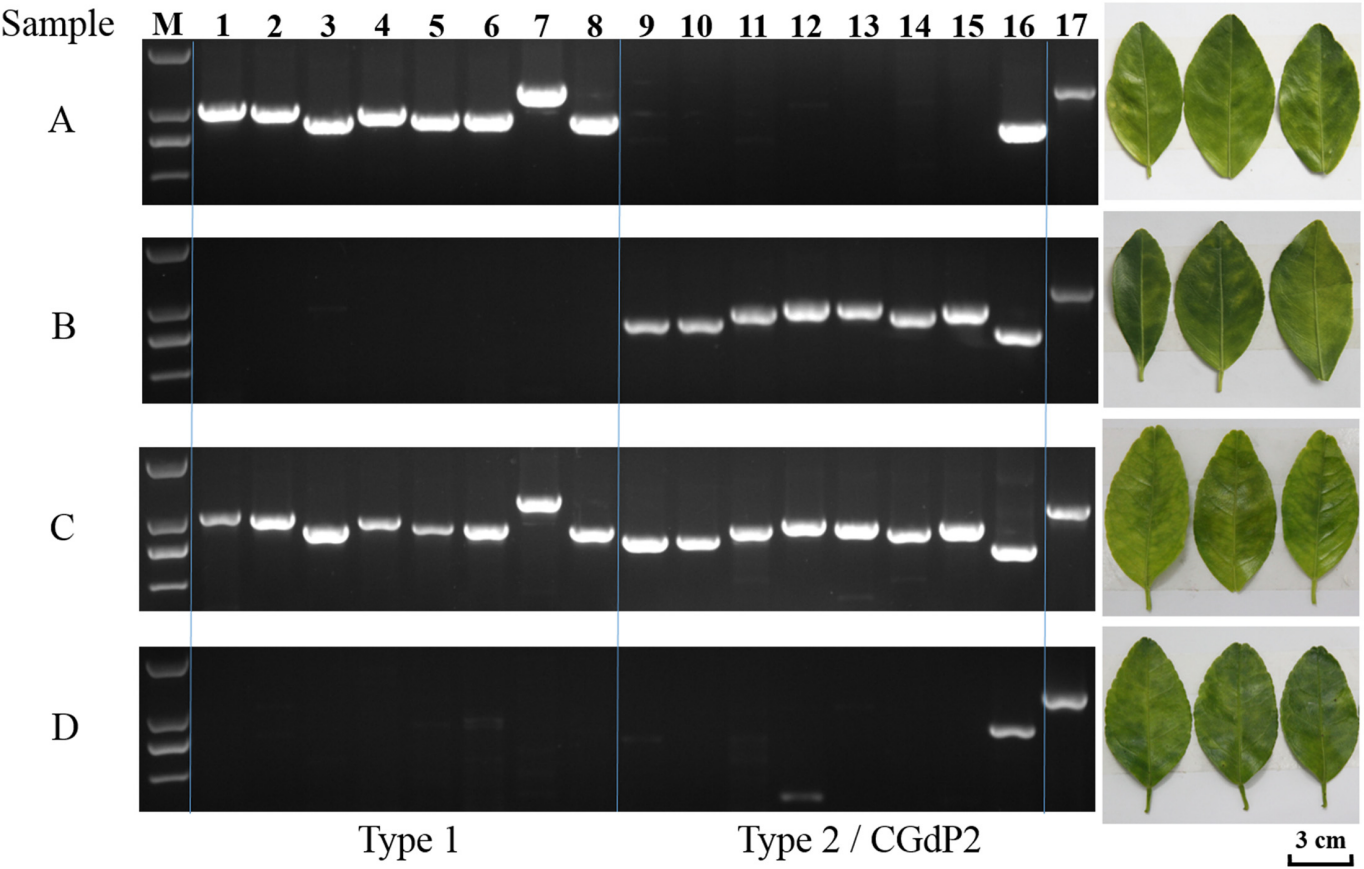
Representative PCR results using prophage type-specific primer sets on samples of “*Candidatus* Liberibacter asiaticus” collected from southern China. (A) Type 1 only; (B) Type 2 only; (C) Type 1 + Type 2; and (D) neither Type 1 nor Type 2. M, DNA ladder (top to bottom in bp: 2,000 bp, 1,000 bp, 750 bp, and 500 bp). Lane 1–8, SC1/Type 1 prophage specific primer sets; Lane 9–16, SC2 / Type 2 prophage specific primer sets; Lane 17, primer set OI1/OI2c for the 16S rDNA region of “*Ca*. L. asiaticus”. Symptoms of citrus leaves where “*Ca*. L. asiaticus” DNA was extraction are on the right. Sample A and D were collected from HLB-infected citrus trees in Guangdong province. Sample B and C were collected from HLB-infected citrus trees in Yunnan and Hainan province, respectively.

As shown in Fig 3, specific primer sets (Table 2) were effective in detecting and defining (6/8 or 75%) Type 1 and Type 2 prophages. Non-target amplification occurred, e.g. sample D lane 12 (primer set 12) and samples A, C, and D of lane 16 (primer set 16) (Fig 3). By design, both primer sets 12 and 16 were Type 2 prophage specific. However, overall prophage type interpretation was not affected. It should also be noted that although sample D is considered as harboring no Type 1 or Type 2 prophage, it is possible that partial Type 1 or Type 2 prophage DNA exist in the bacterial chromosome or a currently unknown prophage.

Among the 86 CLas strains from Guangdong (Fig 2), 71 (82.6%) harbored only Type 2 prophage, likely CGdP2. Adding the 7.0% of Type 1 prophage strains, a near 90% of CLas population in Guangdong harbored a single prophage. Similarly, single prophage dominated each of the four other provinces, although the ratio of the two prophage types varied. Noticeably, strains in Yunnan were dominated by Type 1 prophage, contrasting to those of Guangdong. This is in agreement with the previous observations that CLas population in the high altitude Yunnan Province was different from that in the low altitude provinces such as Guangdong [42,43].

In a summary, a total of 187 CLas strains were collected from five provinces in southern China and analyzed (Fig 2). Among them, 26.74% (50/187) harbored single Type 1 prophage, 63.64% (119/187) harbored single Type 2 prophage. Over 90% CLas strains had single prophage. Only 6.95% (13/187) harbored both Type 1 and Type 2 prophages. It should be noted that in the case of two prophage types detected, it was also possible that the CLas samples might be a mixture of two cell types, each having only a single prophage. Our laboratory recently published three more CLas draft genome sequences, HHCA [23], FL17 [24], and YCpsy [44]. Based on the MiSeq reads mapping to SC1 and SC2, all three CLas strains had single prophage (Table 1).

Our observation of single prophage dominance in CLas is different from the earlier reports of two prophages in CLas strain Psy62 [19], UF506 [20], and gxpsy [21]. The discrepancy may be related to the multiple sources of CLas, that increased the chance of collecting two prophage types. A single prophage was reported in the first report of Psy62 from a single psyllid [13]. In the second report that proposed FP1 and FP2, both psyllid and citrus samples were involved [19]. Similarly, both plant and psyllid samples were involved in the study of SC1 and SC2 [20]. The exception is Strain gxpsy, which was reported from a single psyllid [21].

Another interesting observation was that 2.67% (5/187) CLas strains harbored none of the two prophages. This is the first observation of CLas strains without Type 1 or Type 2 prophages in China, similar to strain Ishi-1 in Japan [25]. The lack of prophage did not seem to correlate to the lack of HLB symptoms (Fig 3). This seems to deviate from the speculation that prophage might be related to bacterial virulence [20] and a peroxidase gene in SC2 could encode a secreted effector that suppressed plant defenses [27]. However, our current understanding of CLas pathogenicity / virulence is very limited.

### A CRISPR/*cas* system

**Fig 4:**
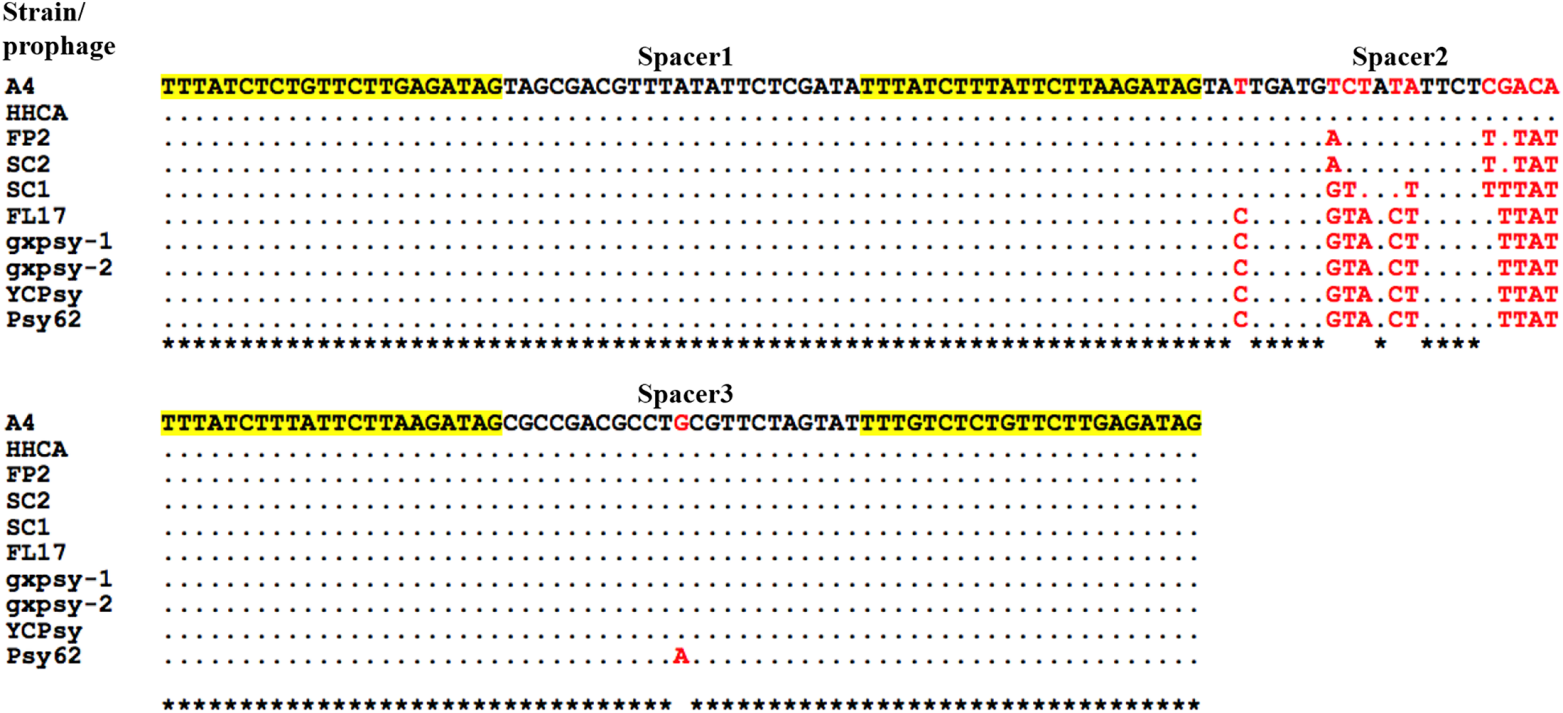
Sequence alignment of a CRISPR (clustered regularly interspaced short palindromic repeats) arrays among ten strains/prophages of “*Candidatus* Liberibacter asiaticus”. Strain A4 was used as a reference. CRISPR repeats are highlighted in yellow. Dots represent nucleotide identity to those of Strain A4. A * at the bottom of alignment indicates identical nucleotides. Nucleotide variations are in red.

**Fig 5:**
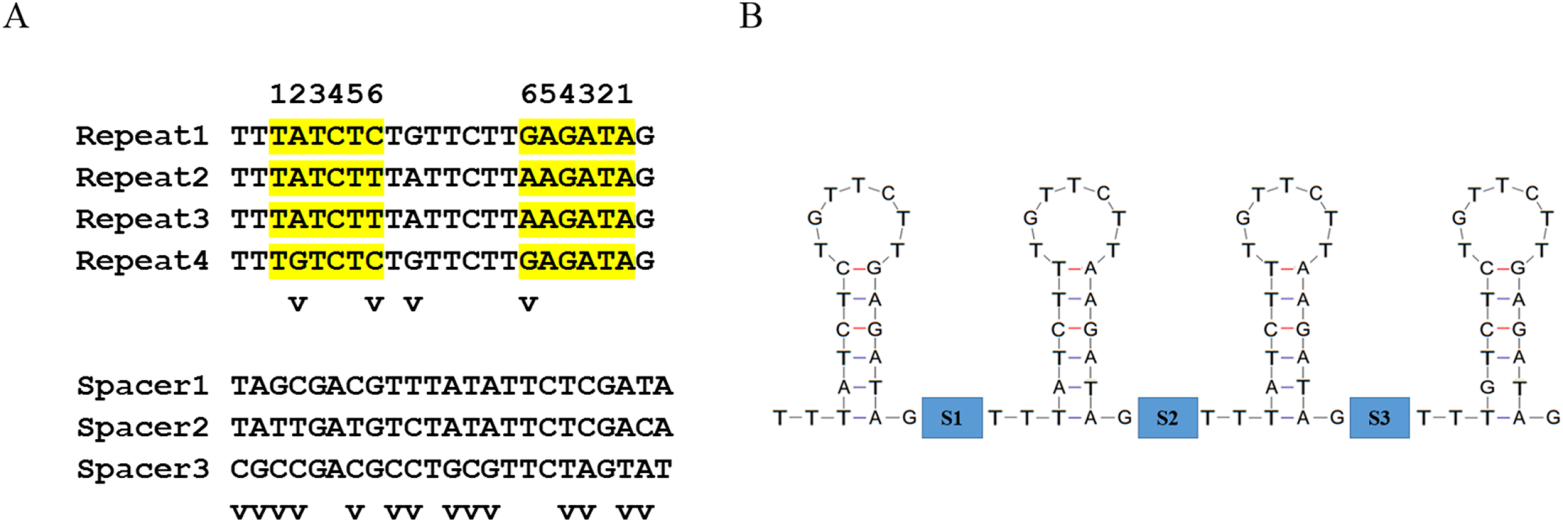
Sequence variations and possible secondary structure of CRISPR (clustered regularly interspaced short palindromic repeats) RNAs (crRNAs) repeats of “*Candidatus* Liberibacter asiaticus” strain A4. A, Multiple alignment of CRISPR repeats and spacers. Nucleotides in yellow involve in stem base-pairing by number matching. Nucleotide variations are indicated by “v”. B, Predicted secondary structures of crRNA repeats using Quikfold on DINAMelt web server. S1, S2 and S3 in blue represent the crRNA spacers.

Analyses of A4 genome sequence revealed seven possible CRISPR arrays (Table A in S1 File). However, CDD search identified CD16_05520 as a putative *cas*4 gene (Table 3), which was 1,682 bp or 4 ORFs downstream of CRISPR candidate 7 (Table B in S1 File and Table 3). This CRISPR/*cas* system was located within prophage CGdP2. The CRISPR array contained four highly similar 22 bp repeats with three heterologous spacers of 23 bp (Figs 4, and 5). Unlike the CRISPR spacers, each repeat had typical dyad structure and capable of forming a stable stem-loop (Fig 5), a characteristic of CRISPR repeat [28]. Repeat sequences were much more homogeneous (82%, 18/22) than spacers (39%, 9/23). No similar CRISPR array was found in GenBank sequence database except for the 10 published CLas prophages (Table 1), suggesting the CRISPR/*cas* system was shared by these prophages.

**Table 3:** Basic information of a predicted CRISPR/*cas* system in prophage CGdP2 of “*Candidatus* Liberibacter asiaticus” strain A4 with comparison to prophage SC2.

Locus name	Nucleotide (bp)	Amino acid	Conserve Domain	Domain ID	Putative Function	Annotation	SC2 locus	SC2 annotation
**CD16_05490**	**2381**	**790**	**Primase_Cterm**	**TIGR01613**	**Primase**	**“*cas*”**	**SC2_gp165**	**DNA primase**
**CD16_05495**^a^						**CRISPR array**	**SC2_gp170**	**Hypothetical protein**
**CD16_05500**	**207**	**68**	**Unknown**	-	**Hypothetical protein**	**?**	**SC2_gp175**	**Hypothetical protein**
**CD16_05505**	**264**	**87**	**Unknown**	-	**Hypothetical protein**	**?**	**SC2_gp180**	**Hypothetical protein**
**CD16_05510**	**372**	**123**	**Unknown**	-	**Hypothetical protein**	**?**	**SC2_gp185**	**Hypothetical protein**
**CD16_05515**	**324**	**107**	**SXT_TraD**	**TIGR03743**	**Conjugative coupling factor**	**“*cas*”**	**SC2_gp190**	**Hypothetical protein**
**CD16_05520**	**1,167**	**388**	**Cas4_I-A_I-B_I C_I-D_II-B**	**cl00641**	**RecB-like nuclease**	***cas*4**	**SC2_gp195**	**Exonuclease**
**CD16_05525**	**789**	**262**	**Bro-N**	**COG3617**	**DNA binding**	**“*cas*”**	**SC2_gp200**	**Phage antirepressor**
**CD16_05530**	**651**	**216**	**DUF2815**	**cl12564**	**Phage related protein**	**“*cas*”**	**SC2_gp205**	**Helix-destabilizing protein**
**CD16_05535**	**2,028**	**675**	**DNA_pol_A**	**cl02626**	**Exonuclease/polymerase**	**“*cas*1 fusion”**	**SC2_gp210**	**DNA polymerase**
**CD16_05540**	**312**	**103**	**VRR_NUC**	**cl22959/pfam08774**	**Endonuclease**	**“*cas*2”**	**SC2_gp215**	**Endonuclease**
**CD16_05545**	**1,386**	**461**	**HepA**	**COG0553**	**Helicase**	**“*cas*3”**	**SC2_gp220**	**DNA or RNA helicase**
**CD16_05335**	**360**	**119**	**LIGANc**	**cl03295**	**Ligase**	**“*cas*”**	**SC2_gp225**	**DNA ligase**

^a^ The open reading frame was annotated to code for a transmembrane protein.

**Fig 6:**
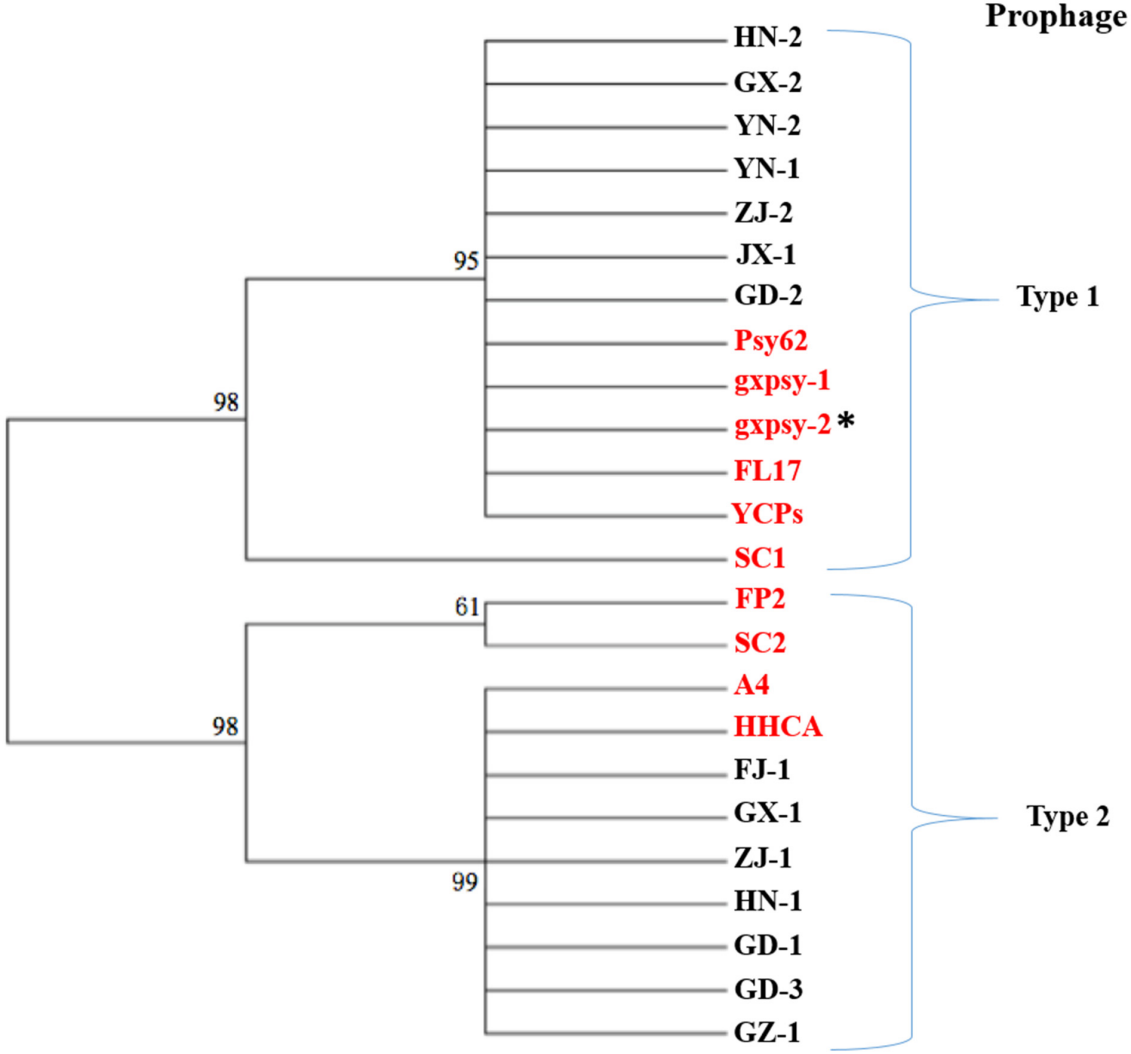
An unrooted phylogenetic tree of “*Candidatus* Liberibacter asiaticus” strains based on spacer 2 sequences of CRISPR (clustered regularly interspaced short palindromic repeats) array. HN, GX, YN, ZJ, JX, GD and GZ represent the CLas strains from Hainan, Guangxi, Yunnan, Zhejiang, Jiangxi, Guangdong and Guizhou provinces, respectively. Published prophages are identified in red. Prophage gxpsy-2, identified by *, is a Type 2 prophage based on sequence mapping results. Numbers at each branch are boostrap values supported in 1,000 replication by neighbour-joining method.

When comparing the 10 CLas prophages from different geographical regions (Fig 4), spacer 1 showed no difference. Spacer 3 is mostly homogeneous except for a SNP in strain Psy62 from Florida. Significant sequence variations were found in spacer 2. Additionally, 14 CLas strains were collected in southern China and their CRISPR regions were compared. Variations were again found in spacer 2 but not in spacer 1 and 3. Cluster analysis showed that variations in spacer 2 grouped along with prophage types, regardless to the geographical origins (Fig 6). BLAST search through virus database with each spacer as a query did not identify any 100% similarity match.

**Fig 7:**
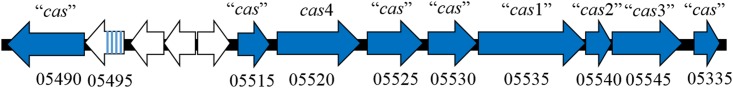
Schematic representation of CRISPR (clustered regularly interspaced short palindromic repeats)/*cas* system in “*Candidatus* Liberibacter asiaticus” Strain A4. The CRISPR repeats are depicted by four vertical blue lines at locus 05495. Open reading frames (ORFs) are represented by arrow boxes with locus numbers listed. ORFs with no predicted functions are indicated by white arrows (locus number omitted for simplicity). ORFs with conserve domains of DNA/RNA enzymes were predicted as “*cas*” genes and indicated by blue arrows. Arrow directions represent ORF directions. The *cas*4 assignment to ORF 05520 was determined by significant match to orthologues in Conserve Domain Database. Genes “*cas*1-3” were proposed mainly based on similar protein functions.

According to annotation, the CRISPR array was found within an ORF CD16_05495. This is not typical among the known bacterial CRISPR arrays which were believed to be intergenic [28]. However, CRISPR array was in the opposite direction of CD16_05495, i.e. the CRISPR sequence itself was not coding. In addition, it was pointed out that CRISPR arrays could be masked by ORFs incorrectly annotated simply based on lack of stop codon in long stretch of DNA sequences [45]. ORFs surrounding the CRISPR array were mostly gene possessing DNA/RNA processing function motifs (Table 3; Fig 7). As discussed earlier, CD16_05520, was highly similar to member of Cas4 superfamily (pfam10926) [28,46,47].

The relationships of other ORFs to *cas* gene in the current version of CDD were less clear. This is not surprising since database of *cas* gene sequences is still in its infancy. Plus, CLas itself is a poorly studied bacterium. A set of *cas* genes designated as *cas*1 to *cas*4 have been regarded as the core genes for a CRISPR/*cas* system [28,48]. Although homologues of *cas*1, *cas*2, and *cas*3 could not be found based on sequence similarity, the CLas CRISPR/*cas* system contained a set of genes possessing functions to those of the *cas* genes, CD16_05535 as *cas*1 for its exonuclease domain, CD16_05540 as *cas*2 for its endoribonuclease domain, and CD16_05545 as *cas*3 for its helicase domain (Table B in S1 File). In another word, the CLas CRISPR/*cas* system possesses all key components to be fully functional.

### CRISPR/*cas* and CLas prophage relationship

It should be noted that most CRISPR/*cas* systems discovered so far are chromosome-borne. It is, however, also documented that CRISPR/*cas* system were carried by phages [49–53]. In *Vibrio cholera*, it was reported that a phage-encoded CRISPR/*cas* system could be used to counteract a phage inhibitory chromosomal island of the bacterial host [53]. In a human gut virome study, Minot et al. [51] demonstrated a strong *in silico* evidence of a phage-encoded CRISPR array targeting another phage.

Our survey from southern China showed that two types (Type 1 and Type 2) of propahges, and therefore inferring two types of phages, coexist (Fig 2). However, for a CLas strain (a HLB citrus tree), single prophage is predominant (90.4%, Fig 2), which could be interpreted as the two prophages were in competition for a host. Considering that the function of a CRISPR/*cas* system was to destroy invading DNA based on spacer information, it can be hypothesized that one pre-established CLas prophage in a CLas cell could use its CRISPR/*cas* system to defeat the invasion of the other phage/prophage DNA. The sequence of spacer 1 or spacer 3 or both could be the target of recognition, although more research such as protospacer adjacent motif (PAM) is involved is needed. Along this line, the role of spacer 2 remains to be investigated.

Having proposed the hypothesis on competitions between the two CLas prophages/phages, we are aware that directly molecular evidence is needed for the ultimate proof of the CRISPR/*cas* system. Yet, this effort could face an even more challenging research issue, the *in vitro* cultivation of CLas that has not been resolved, despite research efforts for decades. Here, we explored the use of *in silico* genome sequence analyses to identify a CRISPR/*cas* system in CLas, which could be related to the observed prophage competitions in southern China. This is the first effort to investigate CRISPR/*cas* system in the genus of “*Ca*. Liberibacter”. In light of the fast advancement of the current *cas* technology[54], knowledge of the CLas CRISPR/*cas* system could potentially be used for gene manipulation of this uncultureable bacterium using the *in planta* (such as periwinkle) cultivation system.

## Conclusions

This study began with the genome sequence analysis on a CLas strain collected from Guangdong Province of China, where HLB has occurred for over a hundred years, and then extended the study to four nearby provinces. The CLas population in southern China was found to predominantly harbor a single prophage. The prophage carried an immunity structure called a CRISPR/cas system. The prevalence of single prophages suggested competition events between prophages for CLas hosts. One prophage might use its immunity structure to defeat the invasion of the other. This is the first finding of an immunity system in CLas. The information will facilitate current understanding on the molecular mechanisms of CLas population variation. Biological information about CLas, the HLB pathogen, is currently in urgent need for development of effective HLB control strategies.

## Supporting Information

S1 Fig Mapping of MiSeq reads of “*Candidatus* Liberibacter asiaticus” Strain A4 to the sequence of prophage SC1 and SC2. Mapping track of A4 Miseq reads to SC1 and SC2 sequence were performed on CLC genomic workbench. Green lines represent forward reads and red lines represent reverse reads. A4 reads covers 57% of SC1 (40,048 bp) and 100% of SC2 (38,997).
(TIF)

S1 File Candidate CRISPR (clustered regularly interspaced short palindromic repeats) arrays and *cas* genes in the genome of “*Candidatus* Liberibacter asiaticus” Strain A4. A list of candidate CRISPR (clustered regularly interspaced short palindromic repeats) arrays detection by the CRISPR Recognition Tool **(Table A).** The nucleotide sequence from CD16_05490 to CD16_05535 in the genome of “*Candidatus* Liberibacter asiaticus” Strain A4 **(Table B)**.
(DOCX)
