# Supplementary figures and images for: Predominance of Single Prophage Carrying a CRISPR/cas System in “Candidatus Liberibacter asiaticus” Strains in Southern China
Source: PLoS One. 2016 Jan 7;11(1):e0146422. doi: 10.1371/journal.pone.0146422 (PMC4711790; doi:10.1371/journal.pone.0146422)

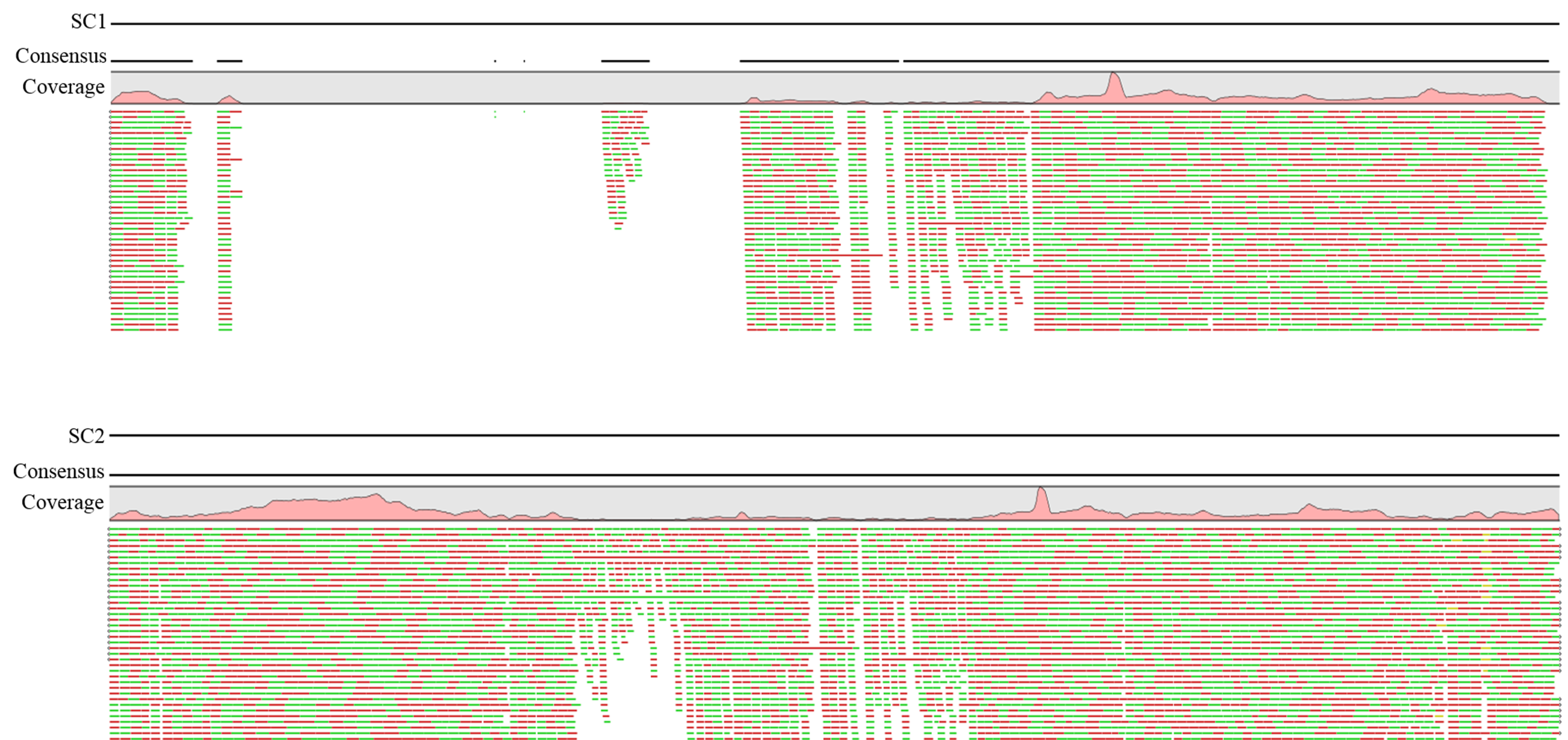

Supplement: S1 Fig — Mapping track of A4 Miseq reads to SC1 and SC2 sequence were performed on CLC genomic workbench. Green lines represent forward reads and red lines represent reverse reads. A4 reads covers 57% of SC1 (40,048 bp) and 100% of SC2 (38,997). (TIF) [file pone.0146422.s001.tif]
