# Supplementary material for: Predominance of Single Prophage Carrying a CRISPR/cas System in “Candidatus Liberibacter asiaticus” Strains in Southern China
Source: PLoS One. 2016 Jan 7;11(1):e0146422. doi: 10.1371/journal.pone.0146422 (PMC4711790; doi:10.1371/journal.pone.0146422)
Supplement: S1 File — A list of candidate CRISPR (clustered regularly interspaced short palindromic repeats) arrays detection by the CRISPR Recognition Tool (Table A). The nucleotide sequence from CD16_05490 to CD16_05535 in the genome of “Candidatus Liberibacter asiaticus” Strain A4 (Table B). (DOCX) [file pone.0146422.s002.docx]

**Table A. List of 7 Candidate CRISPR (clustered regularly interspaced short palindromic repeats) arrays from the genome sequence of “*Candidatus* Liberibacter asiaticus” strain A4 (GenBank Access Number: CP010804; 1,233,514 bp)**

| CRISPR | Position | Repeat sequence (5’ to 3’) | Length (bp) | Spacer sequence (5’ to 3’) | Length (bp) |
| --- | --- | --- | --- | --- | --- |
| 1 | 204440 | ATTTTACCTGCTCAGGAGGCTACGTCTA | 28 | ATAGTCATGGAAAAAGAAACGTCGGTAAAAATCGATAAAACCAAACAAAGTATACGAT | 58 |
|  | 204526 | ATTTTACCTGCTCAGGAGGCTACGTCTA | 28 |  |  |
|  |  |  |  |  |  |
| 2 | 973162 | TTTTTTACTTTTTTGGGAGA | 20 | ATGTCTTTTTCATAACAATATTTTCTTTTCTTGCGCTTTTTGGATCGTCTGTTTC | 55 |
|  | 973237 | TTTTTTCTCGTGTTCGGAGA | 20 | ATTGTTTTTTGGGATTTTCTTGTGATTCTATTAC | 34 |
|  | 973291 | TTTTTTCTCGTGTTCGGAGA | 20 |  |  |
|  |  |  |  |  |  |
| 3 | 1005566 | TCAACTTGATGATCAGGCAGGTTTTCTATTGCAATATC | 38 | GATCTCACTAGCTTGATGG | 19 |
|  | 1005623 | TTTGATTTCAAATCAGGCAGGTTTTCTATTGCAATATC | 38 | GATCTCACTAGCTTGATGG | 19 |
|  | 1005680 | TTTGATTTCAAATCAGGCAGGTTTTCTATTGCAATATC | 38 |  |  |
|  |  |  |  |  |  |
| 4 | 1196416 | GCACGCTTTGACTTCCTCATCGATAAAATGGATAA | 35 | AGAATCTAAAAGAGTCGAT | 19 |
|  | 1196470 | GCACGTTTTGAATTCCTTATAGATAAAATGGATAA | 35 | AGAGTCTAAAAGAGTCGAT | 19 |
|  | 1196524 | GCACGTTTTGAATTCCTTATAGATAAAATGGATAA | 35 |  |  |
|  |  |  |  |  |  |
| 5 | 1197088 | CCTAAAGGACAGACAGGAGATAAAGG | 26 | ACCTCAAGGACCTAAAGGACAGACAGGAGATAAAGGACCTCAAGGA | 46 |
|  | 1197160 | CCTCAAGGACAGACAGGAGATAAAGG | 26 | ACCTCAAGGACCTCAAGGACCTAAAGGACAGACAGGAAATAAAGGACCTCAAGGA | 55 |
|  | 1197241 | CCTAAAGGACAGACAGGAGATAAAGG | 26 | ACCTCAAGGACCTAAAGGACAGACAGGAGATAAAGGACCTCAAGGA | 46 |
|  | 1197313 | CCTCAAGGACAGACAGGAGATAAAGG | 26 | ACCTCAAGGACCTCAAGGACAGACAGGAGATAAAGGACCTCAAGGACCTCAAGGA | 55 |
|  | 1197394 | CATCAAGGACAGACAGGAGATAAAGG | 26 | ACCTCAAGGACCTCAAGGACAGACAGGAGATAAAGGACCTCAAGGACCTCAAGGA | 55 |
|  | 1197475 | CCTCAAGGACAGACAGGAGATAAAGG | 26 | ACCTCAAGGACCTCAAGGACAGACAGGAGATAAAGGACCTCAAGGACCTCAAGGA | 55 |
|  | 1197556 | CCTAAAGGACAGACAGGAGATAAAGG | 26 | ACCTCAAGGACCTCAAGGACAGACAGGAGATAAAGGACCTCAAGGA | 46 |
|  | 1197628 | CCTCAAGGACAGACAGGAGATAAAGG | 26 | ACCTCAAGGACCTCAAGGACAGACAGGAGATAAAGGACCTCAAGGA | 46 |
|  | 1197700 | CCTCAAGGACAGACAGGAGATAAAGG | 26 | ACCTCAAGGACCTCAAGGA | 19 |
|  | 1197745 | CCTCAAGGACAGACAGGAGATAAAGG | 26 |  |  |
|  |  |  |  |  |  |
| 6 | 1222592 | CAAAAAGTTCGTAGCAAGGGGG | 22 | TGGCAAAACACCACCCCCTTAAGACTGAAGGCGGAATA | 38 |
|  | 1222652 | CAAAAAGTTCGTAGCAAGGGGG | 22 |  |  |
|  |  |  |  |  |  |
| 7 | 1225901 | TTTATCTCTGTTCTTGAGATAG | 22 | TAGCGACGTTTATATTCTCGATA | 23 |
|  | 1225946 | TTTATCTTTATTCTTAAGATAG | 22 | TATTGATGTCTATATTCTCGACA | 23 |
|  | 1225991 | TTTATCTTTATTCTTAAGATAG | 22 | CGCCGACGCCTGCGTTCTAGTAT | 23 |
|  | 1226036 | TTTGTCTCTGTTCTTGAGATAG | 22 |  |  |

**Table B. A list of sequences from locus CD16_05490 to CD16_05535 in the genome of “*Candidatus* Liberibacter asiaticus” Strain A4.**

| Locus | Sequence |
| --- | --- |
| CD16_05490 | CTATCTCTTAAAATCTATAATATTGTTATTATCATCCACTGATTCAAAGGCGGGTTTAAGTTTTAATCCTTCGATTACTCTTAAATATCGTCCTCTGTTAGGGCGGGGTTTTTCCCATTGCCTACCCGCTTTAAATCCTTTTTGTTTAAGATTGAGTGCAACTGTTCTCGTTGATATACGTTTTCTATCGTAGTTTAACTCCTGTTCCCGGTATTCACTGTAGCTTTTTGCTAAACTGTGGCTCTCTTCCCATAGATTTTCACCAATATCACAACAATCATCAATCCACGCTTGGTAAGTATCTGTTCCCTGTCTTTCTTCCTCTTTGGCTTTTAAACATACTTCAGGTATATCGACGTCTAGCCCCTTGCTGATGTAGGCTTTAACTCCTTTTAGAAACCATTTCTTCGCTTCTAAGGCGTATTTGGTTTCTAGTTTTTGGGCAAAGGATGCATCCCTATTTGCTATTGGTTTATCGAAAGGGATTACGATGTATCGCCTCCACCATGCATCATCGGGATTTCTAACGAACAGATGTTTGTTAGAGACGATGAAAGGAGTAAAACTAGCGGGTGATTCACTGTAGGTATTACCGTAATTGAGGCGTGCTGTCATGCAATCACCTCCTGTCATTTGTTTTATTTTAGCGGCGTTTATCTCATCATTTTCATTAGTTTCGCTGATAATAACAACTCTAGATCCCATGAGTCTGATAAGGCTAGGGTTTGCTTTACCTGCTTCTGGGGGACGGTTTTGCATAATGTCGCTTGCCTCGGCATTAATGACGTATTGGTTTCCGAAAGCGTATTTAATAAGGTTCATGAGGGTGCTTTTACCACTTCCAAAAACACCTCGAATCTATGTAAGACAATAGTAAAACTGAAGACCCCACTAACAACGCCATTCCAACATCATTACAGCTGAAGACTTATGAAAAAAGCAAAGCTTGTGAAGTATCCTGACACTAGATCAAGAAACTCTTGCGATGACTCACCCTCAACAAAAGGAGTTCCTGTTGATTTGGTAATATATAATTCTTCGGTTGGGGTAATGCGTTGTCCTGTTTCTAAATCTAATATCCCGTCTTTTTCCCCTATGTACCTAAAGTTAGCATCAAAGAAATCCGATGTTATATGAAATTGGCTTTTAGACTCAAGGGCGTTTGCTGTTGATCTAGATTTTGATTGCTCACAAGCTTTGCTTTTTTCATAAGTCTTAAGATATAATAATCTTGGATGCTTCTTAGTGCCGTCTTCGTTTTCTATTGTCTCACAAAGATTAAAGGAATCTTTTTCCTGAGAAACGAGAAACTCCATGATATAACCCTCTATTTTAGCATCTGTAATCCTCCATATATAATGGTTATTCTCGTCCCGTTTATACCATGCTTTAATGTCTGATGCGTAAAGAAAGTGTCCTGATTTAAACACACTGAACATCGCTTTGTTGTAAGCGTCGCTAAACCGAGAAGCTAAAAGACCATCAGGAATTAATTTTCTATGATGATAAAACAGAGACGCAAAAGTAGAACGCTTTTTTGCTGTATCGCCTATTTCTTCAAAATCGAAGGTGTCCCATTTATAATTAAAATTCTCTTCATCATAGGTTGATCCCTGTTTACTCCATCTTCTAGCTATTTCTTTACCTTTACTTGATCCTCTCGTTTCGTGATGAACAGCCATAACAACGGGTATCCATTCATCGTGAGAACCGTTGTAAAATTCTTCGCCAAAACAAGATAAAAAGGCGGTTATTTCTCTATTGGTGTACTGTCTATTATTGTTGTTAGTCCACGTTTTAGAGGGTATAATCGATTTTTTATCTTTTACCAGTGGCACGGTAATCTCTTGAAAAAACTTAAAAAGATATTCAACATCTTCTTCGGAAAGCAAAGGGGTATCTTCCACTTTAAATCTATGGGGCGGGGTCGTCCATGTGTATTCTTTTTTTGTTTTAGGGTGGATATTATAGGCAACGAAGTATTGACCACAACCCAAAATATCAAGATGCCCTTGGGTACTTTCAGTCGTTTTCTTCTTCTTAATCCCTTCTTTATTCATTCGGAAAGGAATAAGAATTTTGGGCTTTTGCCCTATTCTTACGATCGGCGTTCCATGAAGAATCTCAAAAGTATCTTTAAAAGTATTGGCGGTTTTTTCGTCTTTTGAATCAATATCAAAGGCGTAAAGTGGCTGTTCCCCTACGCCACAGACAAACCCAAAGCCACAAGCAGGAAGTTTGTCGATTTTCTCACTTGAAAGTAGTTGTTCTTCCCACTTACCCAATCGCTGTGGTCTTTTATCCCCTAAACGCAAAGGGATGAGCTTAAACCCATTATGAATGGCTTGTTTAGCCTGTTCCTTCCATTGCATTACCGGCAT |
| Intergenic | None |
| CD16_05495^a^ | TTAACTTCTCTTATAAATTAGTTCTTCTATAGGTTTGGCTTTCAGTCTTGCTATCTCTCTTTCTAACGCTTCGATCTCAGCTTTTGTCATGCCCATTGGCTCTTCTGGAGCTATCCAGCTATCTTTTGAATATAATTTCCGGTAAGACTGACGGGCTTTTTCTCTCATTTTATCTCTGTTCTTGAGATAGTAGCGACGTTTATATTCTCGATATTTATCTTTATTCTTAAGATAGTATTGATGTCTATATTCTCGACATTTATCTTTATTCTTAAGATAGCGCCGACGCCTGCGTTCTAGTATTTTGTCTCTGTTCTTGAGATAGTAGCGACGTTTATATTCTCGTCTACACAACATCCTTTCTTCAGGTGTTAAAACTTTTCTTTCCAT |
| Intergenic | AGTGACGCACTCTCTTT |
| CD16_05500 | TTAACTGATCGCTTTAGCGAAAGCACTAATCACGAACTCTTTATACTCTCTGAGCTTTTCTTCGTCTGTTGTTTCCCCAGCTACTGAGACCACAACCGAAAGACGGGCGATATTGTCTTCCTTCATCCAGCCATCCGCTCGAAAAGCGGCGGTGCGTCCTCTTTTTCGGGTATCGAAGTGTTTAACCCCTAATTTAACTGTGTTCATTGACACAC |
| Intergenic | None |
| CD16_05505 | TCATTGACACACCTCACACATATCGTCATCGGGATTATCGCTTCTGCTACTATTTTTAATTCTTTCCCTTTTCACCAGCTCATCAAGGCTTTATCCTCAAGGGCTTTAAAAGCTCATTAGCACTGTCTTTAGCATCTAGCTCAATGTGTCTTAAAATGTCTTCCGATACCGTCCCTAAACCAAGACGGAGAATTAAAACGCTATCCTGATCCGCAATACGACGGGTTAAAACGTCGAATTTAGTTCCATTCCATGGTGTATTTGTCAT |
| Intergenic | CTTAAAACACTCCTTATTCTTTGGTTGTGTTTGATGATCCTTTTATAGCATAGGCTGTTAAAGGTGTCTAGAAAAATAATTAACATATTTTCTACATTTTTTCTTGACTAAGTAGAAATTATGTTAAATAATACCCCCAACAAAAACATAAAATAAAATTCAATTAGGCTTAAATCGCAAACCAGGGAGCGTACAG |
| CD16_05510 | ATGGGAGCATTAAAGAATCATTTTCATGACGAGATTAACGAGAACTTTTACTTTCATAGTCATCCGAACGCAGATCCAGATATAAGTATTGAGATGCAGATTAGCGAGAATCAGCGGTATTTGGATGAAGAGATATCGCAGTGCAATGCGGTAGTTGATGTTTTTAAAAGGTCTGATTCCACCATTTTAGACAAGCTAGATGCGGTGGATGACCTAAAAACTTATATTTCTCTTCTGCAAGCTACGGCTAAAAATCTCAAGTCATTGCTTAAGGAATATTGGGAAGAGTCTTTGGACGGAGAGGACGACGAAGAGATTTATGAGCATCCTGATCAAGAGCATAGAGAAGATTATTATGCCAATCAGATTTAA |
| Intergenic | GAGATCCCCAGTGATGGGTTCAGGGGGTGGATGTTTATATCTCCGTCCATCCCCAACAAATAAAAGTGTAAGAGCGGGAGCGAGTGAGG |
| CD16_05515 | ATGAGCGTACATTTTATTGAAGAGGCGGTTAAAGCCAAGGACATACCACAGTTGTTAACTTTTCTTTCTTTGATCACTCAAGGGTTGCAGGAGGCTTTAATCACCCAAGATGTTAAAGCCGTTGAAGCGGTGGATCCAGACCTCAAGAAGAGAGTCACGGTTTTAGCTATATCCTACATGAAGAGATGTGGTGATAAGGGGAAATCGCAGTTTTTATCCGAGATTTTGGTCCCTGCTTTAGGAACGCATAAAACTTTTGTCGATTGCACAGATGAGGATTTTAGATTGGTGGAAGCGAAGTTATTGGAGCAAAGCGATGCATGA |
| Intergenic | TTGCTTTTTCACCATTGGGGTTCTGATTTTTATTGTCGTTATGGTTTATTTATCACTTCCCGAGAGATCCTGACGATACCACCCCCGCTTAAACGAGAGTTGAATAAATTTAAGAGAGTGAAGATTA |
| CD16_05520 | ATGGCACACCATGCATTTTTATCGGCTTCAAGCAGTCATCGTTGGCTAAAATGTCCTATAGCCCCGACGCTTGAGAGCAAAATACCTCAAACAACAAGTATATACGCCTCTGAAGGCACTTTTGCCCATAATCTTTTAGCGCATTGCCTCGAACAAGGCGTTGATGCAGAGACAGTTTCTCATCAAAAGCTGACTTTTGAGAACGATACCCGCATTGTTGATACGGAAATGGCGTCTAGTGTCTCGATGGTTTTAGCGTATGTCCGCACTTTTTCTGGACCATTTTTATCGGAAACCGAAGTTCCCCTTGAGCCTTTTACAACGGAACCAGGGGCAACCGGCACTGCGGATATCCTTATTTTTAATAATGCCCAGTGGATCATTGCCGATTTTAAATATGGGGCTGGCGTTCCTGTTAATGCTGAGAACAACCCCCAATTGATGTTGTATGCCTGTGGTGCATTGCACTTATTTGCAGATGTCATGGGGAAACCCGAAACTATCACAATGACTATTCTCCAACCCCGTATTAAAACAAGTGATCCAATCAAGGAATGGACGATATCTGTAGCTGAATTGGAGGCTAAAGCCCAAGAGTTCAGAACCAAGGGGCAACAGGCGTTAAAACTTAAGACTAAACGATTTATTCCCTTTGATAGTTATGGAGTGGATGAGAACGCTTGTCGATTTTGCCGAGCTAAAGTTCGTTGTCCTGCATTGAGTAGACATGTGTTGTTAGAAGCGACAAAAGACCCATCAACCAACACAACTGTTGAATTATCGAAAGCCTATTCCTCGATTTCGTTGATCAAGAGTTATGTGAAGGCGTGTGAAGATGAGATGTTTAAACGGTTGAATGCTGGGGATGAAATACAAGGGTATCAACTAGTCGAAGGACGCAAAGGAAATCGAAGTTTTAAGGATATTAACCGAGCGCAAGAGCTGTTGACATCAGTTTTAGGGGAAGAAGCGTTTAAGCGAATTCTCAAAACTCCTAAAGAATTGGAGCAATTGTATAAGGAGCAAAAGGTTTCTGATGAATTTTGGGAAGAGTTGCAAGAGTTGATCACGAGAGGCGACGGCAAGCCTGTCATCGCCCCCCGAGATATACCAACAAACAAACAAACCCAAAAATCGCAATTAAGCGAGTTCGAGGTAATAACATAG |
| Intergenic | AAAGGATCAAAGTGATGATAAATATAACACCTTTTGAAGAATTCAATATAACGAGCGATATACCAGAAATAACGAAAGAAGTCAATAGACTACGTAATGTTTAACGATATCGAAGTTTCACGGCTCAAATTTGAGCCGTCAGATTTGGAAAGTATAGAAAGGATCAAACG |
| CD16_05525 | ATGAGCACTATAACCCCTTTTGAATTTGAAAGCAACAAGATCCGTACTATTGTGGATAAAGACCAGAATATATGGTTTGTAGCTAAAGATGTCGCTGTAGCATTGGGGTATGAACGTCCTAATAATGCGATAAACGCTCATTGCAAGGGAGCCCTGAAACGAGGCACCCTTAAGACCGAGGGCGGAACTCAAAAAGTTCGTATCATAGCAGAACCTGATGTTTACAGGTTACTAGTTAAAAGCACGTTACCATCAGCACAGAAGTTTGAGCGTTGGGTATTTGAGGAGGTGTTACCTACTCTTCGCAAAACAGGCAGTTATTCGGTTGAAGCACCGAAGCTTAGAGCCACTTCAGCGAGTACAGTTTTAAGAGTTCACAAACATCTTGAAGAACTAGCCAAGCAAGCAGGATTAAAAGACAATCAACTTTTGTTGAAGGTTAACCGAGGCGTTACGAAGATAACAGGTGTTGATCAGTTAGAGGCTATGGATATCAAACACCTACCCTCTTCCGATAACGACGAATATTTAACAATTACCCAAATAGGGGAAAGGCTTAACCCACCTCAGAGAGCGAGATTTTTAAACAAACTTCTTCTTAAGCGAGGACTGCAAGTTAGCAAGGTCTCAGGTGGCTATATACCGACACCTAAAGGCGAAGAGTATGGAGGTAAGATGTGTGATGTGCCAATGCACCATGTTGAAGGCTCAACGCAATCTCTTAAATGGAATTCGAGCCTACTCGTTCCGTATCTTCAAAACGAATTTAATAACAATCAACACTTATAA |
| Intergenic | TATGAAAGGACTTTAAAA |
| CD16_05530 | ATGCAAAAACTAACGGTAAAAGGACGCCTATCGTACCCTGCTCTTGATACGAAGGTGCGTATGAAGCTCCCTGATGGCTCAAGTGTTGAGCACTACGGCTGTGATATCGTCTTTCCGAAAACGGATACGAAGCAGATTAACGCTGTTGAGGCGTGTTTAAAGACCGCTGTTACCGAGATATTTCCGAATGTCTCTCCGGATGCGTTTTTGTCGGCGGTTCGAAGCAAATCGGAATCCCGTGGGGTTTTGAGGGACGGCGATGCGAAGATCGCTTCTTCTCACAAGCCTGAGAATTACACACAAACATACACAGACAGCGTGTATATCTCGGCGAAGAACAAATATGTTCAACCGTTACTCGTGGATCGTCAAGCGCAACCTGTGAGCAATCCGAGGGAGGTTTTTTATGCGGGGTGTTGGGTCATTGCCAAACTCAACATCGGGGCGTATGAGCTTGATCCGTACAAAACCAAGGGTTTCAGTTGCACGCTAACGGGCGTTCAGTTCTTCAAGCATGATGAGCGTTGGGGTGCTTCCCCGAAATCGGACACTTCGGAGTTTAAGGATTACGGCGAAGAGCAAGACTCCGATACCTCGGTCTCAAACTTTGCCTCAGCGGAGGTGGACTCCGACGCTCTGCCGTGGAACTGA |
| Intergenic | TCA |
| CD16_05535 | ATGTCAAAGCTGTTTATCGACATTGAAACCCGCAGTCCTCAACCCTTGCCTAAGGTAGGGGTTTGGGCTTATGCGGAGCAGGCGGTTATCACTTTATGTGCGTATGCATGGGACGATGAACCTGTGAAGTTGTGGGATAGGACTGAACAATCTGCTATGCCATCGGATCTTCTGCAGTACTTAAGAGATGAAACGGTGATGTGTGTTGCACACAATAGTCTGTTTGAACGGATACTGTTTAAGAAGACATTAGGTATTGATATTCCATCTAAACGTTGGATTTGTACGAGTGTTTTAGCCCGCACGAATGGTTTACCCTCTTCGTTGAAAAACGCTTGTTTAGCACTAAATTTTTCTGAACATTTAACCAAAATGGAAGAAGGAAAAGCTCTTATCGCTCGTTTTTGCAATGGATCGATTGATTCCCCTCCGTATGACTGTACTCGTGCTAATCACGTTCAAGCATGGCAGTTATTTGGGGAGTACTGCAAACGAGACGTTGAAGCCACTAGAGAGTTATTTAAGCGACTTATTCCTTTATCTGATGGGGAAAGGGATTTATGGCTTTTAGATCAAACGATTAATGATCGGGGCTACAGGATAGACTTAGATTTAGTTTTGAAGCTTCAAGAGCTCATAGCCCAAGAGCGTAAGAAGTTAGATGAAGATATCGTTAAATTGACAGATGGTATTATACGCTCTTCTCGTCAAACGTATACTTTAAGGATGTACCTATTTCTTATAACGGGAATAGATTTGGTTGATATGTCGGAGGGTACTTTAAAGTCTATTCTGTCTCATTCTAACATCACTCAATTGGCTAAAGACCTGATTTTGAACCGCTTAGCATCATCGGGATCAGCAATTTTAAAACTCAACACTTTATCTGAGGCTGTTAGTTCTGATGGGCGTCTTCGAGGGACTTTACAGTTTTATGGTGCGAGCAGAACGGGTCGATGGTCTGGATGTGTGTTTCAACCTCAGAATCTTCCTCGTCAAGAGCGATCGGAGGAAATATTGACACAGACTATTCAAGCTTTAAATCGAGGGGAAACTATTGCAGATCCGTTAGGTTTGGCTAGTGATTGTGTCCGTTCTTGCATCATAGCCTCAAATGGCAAAAAGCTTGTTGTTGCGGATTTAGCAGGGATTGAAGCCCGGGTTTTGGCATGGATTGCTGGTGAAGACTGGAAAATCAAAGCTTTTGCAAATGGTGAAGACATCTATGTTACAACTTATGCTAAAGCGTTTAACACCCCCATTGACAAAGTGAGTAAAGAACAAAGAGCTATTGGGAAAGTTATGGAATTAGCCTTAGGGTATCAAGGTGGGGCTAAGGTGTTTAAGACAATGGCATCTCATTGTGGATTAGATCTACAACAGTTTAGTCAGAATGTCAAAAGCACCTCAACTTTTGAAGATTGGGAACAAGCGGAATCTCATCATCTTTGGATGCAAGATCAATATCCCGAATTTGCGGTGAAGGATAAATTGATAGGCACGGCTTGTGAACTTGTTAAAAAGGCTTGGCGGGCTAAACATCAAGGTGTTCTTCAACTATGGAAGGACTTAACCGAGGGTTTTGCATGTGTAGTGCAAGAAGGAGGTTCAATATCTGCTCGAAGGGTTGCGAATGTTCCCCGTCTTGTGATGAGGAGGCACAAACGAGACGTTCACATTGTTTTACCTTCATCAAGAAGGCTTGTTTACAGTGATGTAAAGGGCGATTGTTCTTATCTCAATACTGCCACTTCACAGCTCATGAGAGAGAGAACGTATGGTGGCAAGCTAACAGAGAATATTGTTCAAGCCATCAGTAGGGATATCCTTTGCGAGGGCATGAAAAACGCTACTAAAAATGGCTATGACATAGTTTTAACGGTTCATGATGAGATTGTTAGTGAAACTCCTGATACTCCTTACTTTAGCGTTGGGACCTTATGTTCTTTGATGACAAAAAATCCCAGTTGGGCAAAAGGTTTGCCCCTCAAAGCAGAAGGTTATGAAGCGAAAAGGTATCGCAAATGA |
| Intergenic | None |
| CD16_05540 | ATGAGTTTTTATTCTTTCCATTATCAGACAGAAAAAGACGTGGAAAAGCGTTTAGTGACAGGGGCAAAGAAACTTGATTGTTGGGTTCGTAAAGCATCGTTTGTAGGACGTAGAGGTTGTCCAGATAGATTAATCATCACCCCTAATGGGGGTTTATGGTGGATTGAAGTCAAAAAGCCAACAGGAAGATTATCACACCAGCAGATGAGTGAAATAGAAGAACTACGACGGCGAGGACAGCGGGTTAAAGTTCTGATCTCTATTGAGGAAGTAGATAACTTTTTGGAAGAATTAGCATGCACCTCGTATTGA |
| Intergenic | None |
| CD16_05545 | ATGCACCTCGTATTGAAACCCCATCAAATTGTAATGGTTAATTGGCTCTTAAGTCATGACAGATGTGCTTTGTGGGCTTCGATGGGTTCGGGAAAGACGGTAAGTGTCTTATTCGCCTTATCCACTATTAAAATACTTGATCCTCGTCCTGTCCTTATTATTGCACCCTTACGAGTTGCCCAATATGTTTGGAAGGATGAGGTGGAACGCTGGTCAGCATTTAGTGATATGACTGTTTCATCTCTTATTGGATCGGAACGACAAAGAATAAAGGCTCTCAATACTCCTGCTCACCTTTACATTATTAATTTTGAGAATATCCCTTGGTTAGTCAAAATGAAGCTTGATCATTGGGATTTTGCAACGATTGTTGTTGATGAAAGCACAAAGTTGAAATCTTTTAGAACCCATCAAGGAACAAAGCAAACGAGAGCTTTAGGGAAAGTAGCTTTTAGCAAAGTTGAACGTTTTATTGAGCTCACAGGGACCCCTTCGCCTAATGGCTTAATCGACTTATGGGGTCAAATATGGTTTTTGGATAAAGGAAAACGTTTAGGACGTGTCTTCCAAAGCTTTGTGGCTCGCTGGTTTAATACAACACAAATCGGATCTCATATTGGAGCGGTACGGTATACGGCTAAAGAAACCGCCCAAAAAGAGATAGAAGCGCAATT |
| Intergenic | ATCTGATTGTTGTTTATCATTAGATATCGCTGATTATCAAAATATTGATAAACCTATTCTCATCACTAAAAAAGTCCCATTACCCCAACCGGTGATGAAGCAATATCACAAGTTTCAACGAGAGTTGTATTGCGATCTTCAAGGAGAGAATATTGAAGCGTTTAATTCCGCTTCTAAAACTGTCAAGTGTCTCCAATTAGCCAATGGTGCGGTGTACTACGACGAAGAAAAACATTGGAAAGAAGTCCATGATGAAAAGATCAAAGCGTTAGAAGTCATCATTGAAAAAGCCAATGCCGCCCCTATTATCGTTGCTTATCATTTTAATAGTGATCTTGCTCGATTGCAAAAAGCATTTCCCCAAGGCCGAACGTTGGATAAAGACCCTTGCACTATTCAAGAGTGGAATGAGGGGAAAATTCCTTTGTTGTTCGCACATCCAGCGTCTTGTGGCCATGGCTTAAATCTCCAATATGGCGGGAACATTTTAGTCTTTTTCTCCTTGTGGTGGGATTTAGAAGAACACCAACAGATGATTGAACGCATTGGGGTCACGCGTCAACGACAAGCGGGATTCAAAAGAGCGGTTTTTGTCTATTATTTAATTGCCCAGAACACCATAGATGAGTTGGTTTTGCAACGATTGCGAACTAAATCCACTATCCAAGACTTGTTGTTAAACGCTTTAAAAAAGGAGACTATCC |
| CD16_05345 | ATGTATAATTTTGACAGAGTTTTTAGATCTAGTAAATTTGAAAACGAGCATAATATTACTCCTGCCCAATGGAAAAAGCTTTTAACGCTTGAAGCAAAGTTTCTTCCAAATAAGCGCGCCCTAGAATCATGGCTCGACAAAGCAAAAAAGGTCACATCGCTGTCGAAGGGAGAAGCGATGATTGAAGTTGAGTATCTGGTTAAAATCGCTCTCCATCATCAGAAGTGGTATTATCGGTTGGACGACCCTCTATTTACTGATGGGTTATATGATCGCGTATCAGAGCGACTCGACGCATTACAAGAGCAATTCCCTGAGCTTTTTGATGAGGATCATCCATGGAACACCGTGGGGTATTAA |

^a^ The repeat of CRISPR array is highlight in yellow and the spacer is in red.
